# Association Between Donor Human Milk Initiation Timing and Necrotizing Enterocolitis, Mortality, and Feeding Outcomes in Very-Low-Birth-Weight Infants: A Multicenter Retrospective Cohort Study Using Two Japanese Registries

**DOI:** 10.3390/nu18121855

**Published:** 2026-06-09

**Authors:** Yuka Sano Wada, Hiroki Den, Motoichiro Sakurai, Yuki Tani, Jun Shindo, Masafumi Miyata, Shigeru Nishimaki, Katsumi Mizuno

**Affiliations:** 1 Division of Neonatology, Center for Maternal-Fetal, Neonatal, and Reproductive Medicine, National Center for Child Health and Development, Tokyo 157-8535, Japan; 2 Department of Hygiene, Public Health, and Preventive Medicine, Showa Medical University Graduate School of Medicine, Tokyo 142-8555, Japan; 3 Department of Pediatrics and Neonatology, Kameda Medical Center, Kamogawa 296-8602, Japan; 4 Department of Pediatrics, Nara Medical University, Kashihara 634-8521, Japan; 5 Neonatal Department, Tokyo Metropolitan Children’s Medical Center, Tokyo 183-8561, Japan; 6 Department of Pediatrics, Fujita Health University School of Medicine, Toyoake 470-1192, Japan; 7 Department of Pediatrics, Yokohama City University, Yokohama 236-0004, Japan; 8 Department of Pediatrics, Showa Medical University Graduate School of Medicine, Tokyo 142-8555, Japan

**Keywords:** donor human milk, necrotizing enterocolitis, very-low-birth-weight infant, enteral feeding, neonatal nutrition, Bayesian regression models

## Abstract

**Background/Objectives**: Early enteral feeding in preterm infants remains controversial because, despite promoting intestinal maturation, concerns about necrotizing enterocolitis (NEC) persist. Donor human milk (DHM) is recommended when the mother’s milk is unavailable, but the optimal initiation timing is unclear. This study evaluated the association between DHM initiation timing and outcomes in very-low-birth-weight (VLBW) infants in Japan. **Methods**: This multicenter retrospective cohort study used data from a national human milk bank registry (2018–2023) and the Neonatal Research Network of Japan (NRNJ; 2022). Infants with birth weight <1500 g were categorized by DHM initiation timing (≤24, 25–48, or >48 h), with infants in the NRNJ cohort serving as the comparison group. The primary outcome observed was NEC incidence, including all stages; secondary outcomes included in-hospital mortality and time to full enteral feeding. Bayesian regression models were applied. Detailed feeding data were unavailable in the NRNJ cohort. **Results**: Among 2962 infants, NEC incidence was low across groups. NEC occurred in 1.31% of the ≤24 h DHM group and 1.83% of the NRNJ group, with mortality rates of 4.53% and 6.23%, respectively. Although NEC incidence was numerically lower in the ≤24 h DHM group, estimates were imprecise because of limited events. Early DHM initiation was associated with lower in-hospital mortality and earlier full enteral feeding, particularly in infants with <1000 g birth weight. **Conclusions**: Early DHM initiation was associated with lower mortality and earlier achievement of full enteral feeding in VLBW infants without elevated NEC risk. However, because detailed feeding information was unavailable in the comparison cohort, these associations should be interpreted cautiously. Given the observational design and heterogeneous nutritional exposures, further prospective studies are warranted.

## 1. Introduction

Very-low-birth-weight (VLBW) infants require a delicate balance in early nutritional management. Optimal nutrition is crucial for growth, neurodevelopment, intestinal maturation, and immune regulation [1,2]. Although early enteral feeding may promote gut maturation and reduce the duration of parenteral nutrition, concerns about necrotizing enterocolitis (NEC) and feeding intolerance persist, and randomized trials and meta-analyses have revealed inconsistent findings regarding its clinical benefits [3,4,5]. Consequently, clinicians frequently delay enteral feeding; however, extended fasting can induce intestinal mucosal atrophy, impair gut motility, and increase the risks of bacterial translocation and infection [6,7].

Donor human milk (DHM) acts as the recommended alternative for preterm infants when the mother’s milk is unavailable immediately postdelivery, frequently due to maternal separation or delayed lactogenesis [8,9]. Several studies have demonstrated that DHM decreases NEC incidence compared with formula feeding, reinforcing its use as the preferred substitute [10,11,12]. However, previous studies have reported inconsistent findings regarding the effects of early enteral feeding on NEC and other neonatal outcomes [3,4,5,13]. Nutritional exposure in clinical practice is heterogeneous given that infants may receive their mother’s own milk, DHM, formula, or mixed feeding and that feeding practices may vary across institutions. Moreover, DHM differs from the mother’s milk in terms of composition and processing, including pasteurization. The early postnatal period represents a critical window for intestinal–immune development, during which exposure to human milk–derived bioactive components may modulate inflammatory responses and enhance outcomes, including NEC and mortality [8,14,15,16]. Despite the established benefits of DHM, most previous studies have focused on its availability or overall use rather than on the timing of its initiation. Whether initiating DHM within the initial 24 h of life confers additional protection against adverse outcomes remains largely unexplored. Moreover, evidence on how the precise timing of DHM initiation impacts neonatal outcomes in VLBW infants is scarce.

In Japan, nutritional management practices for VLBW infants have remained predominantly unchanged from 2018 to 2023, with many NICUs commonly practicing gradual enteral feeding advancement and preferential use of the mother’s own milk when available. However, no standardized national guidelines exist regarding enteral feeding initiation timing or DHM use. Notably, DHM programs were relatively recently introduced, with the establishment of a human milk bank system in 2017, potentially impacting clinical practices during the study period.

In Japan, a nationwide human milk bank registry systematically records DHM initiation timing, facilitating time-based analyses at an unprecedented resolution. Leveraging this unique dataset, we aimed to investigate the association between DHM initiation timing and key neonatal outcomes, including NEC, in-hospital mortality, and time to achieve full enteral feeding. We hypothesized that earlier DHM initiation, particularly within the first 24 h after birth, might be associated with improved clinical outcomes among VLBW infants.

## 2. Materials and Methods

### 2.1. Study Design and Data Sources

This study encompassed a multicenter retrospective cohort of VLBW (<1500 g) infants in Japan and utilized two complementary data sources.

The DHM cohort was identified from the national human milk bank database, which prospectively registers all infants receiving DHM. Infants born between 2018 and 2023 were eligible. The database contains comprehensive perinatal information, including DHM initiation timing, enabling the classification of exposure by postnatal age.

The comparison cohort was drawn from the Neonatal Research Network of Japan (NRNJ) registry, which is a nationwide prospective database established in 2003 that collects detailed clinical information on preterm and low-birth-weight infants across Japan. Standardized data on perinatal characteristics, neonatal morbidities, treatments, and outcomes until discharge are included in the registry. For the present analysis, data on infants registered in 2022 were used. However, comprehensive data on early nutritional exposure, including enteral feeding type (mother’s own milk, formula, or mixed feeding) and feeding initiation timing, were inconsistently available in the NRNJ registry. Therefore, infants without documented DHM use were categorized into the non-DHM group irrespective of specific feeding practices.

### 2.2. Participants

Infants with <1500 g birth weights who were registered in either the DHM database (2018–2023) or the NRNJ registry (2022) were eligible. Exclusion criteria in the DHM database were missing information on DHM initiation timing and receipt of formula before any DHM feeding. In the NRNJ registry, infants recorded as having received DHM or those already included in the DHM database were excluded.

### 2.3. Exposure and Comparator

#### 2.3.1. Exposure

The receipt of DHM was the exposure of interest. Infants registered in the human milk bank database from 2018 to 2023 who received DHM were categorized into the DHM cohort. The timing of initial DHM administration was recorded in hours after birth, and infants were categorized into three exposure groups: ≤24, 25–48, and >48 h after birth.

As this study primarily aimed to evaluate the effect of early DHM initiation, the main analyses centered on comparisons between infants for whom DHM was initiated within 24 h and those for whom DHM was initiated at 25–48 h after birth. Infants for whom DHM was initiated after 48 h were considered a clinically distinct group reflecting delayed feeding initiation and were therefore included for descriptive and supplementary analyses. Comprehensive data on feeding practices before DHM initiation in this group was not systematically available.

#### 2.3.2. Comparator

The comparison group comprised infants registered in the NRNJ in 2022 without documented DHM use. To prevent overlap between cohorts, infants with documented DHM exposure in the NRNJ or duplicate registration in the DHM database were excluded.

### 2.4. Outcomes

The primary outcome observed was NEC, recorded in both databases as a binary variable (0 = no NEC, 1 = NEC present), including all stages of NEC. NEC was clinically diagnosed by attending neonatologists at each participating center and recorded in the registries. Stage-specific data were unavailable. Secondary outcomes encompassed in-hospital mortality, defined as death before hospital discharge, and age in days when full enteral feeding was achieved, defined as the first postnatal day on which a ≥100 mL/kg/day enteral intake was recorded.

### 2.5. Covariates

All adjusted analyses included the following prespecified covariates: sex, gestational age in days, birth weight, and 1 min Apgar score. These variables were identified before DHM exposure and were available in both databases. Covariates were selected a priori based on clinical relevance.

In the sensitivity analyses, we further adjusted for small-for-gestational-age (SGA) status, mode of delivery (cesarean section), antenatal steroid exposure, and treatment for patent ductus arteriosus. These variables were included as they may reflect perinatal condition and clinical management that could impact feeding decisions and neonatal outcomes.

SGA was defined as a birth weight below the 10th percentile for gestational age and sex.

### 2.6. Statistical Analysis

Baseline characteristics were compared across four groups (NRNJ, DHM ≤ 24 h, DHM 25–48 h, and DHM > 48 h). Standardized mean differences (SMDs) were calculated for comparisons between each DHM group and the NRNJ group, which served as the reference. Continuous variables were summarized as medians (interquartile ranges) and categorical variables as counts (percentages). An absolute SMD <0.10 was considered to indicate negligible imbalance.

To estimate the association between DHM initiation timing and binary outcomes, including NEC and in-hospital mortality, Bayesian logistic regression models were employed.

As this study mainly aimed to evaluate the effect of early DHM initiation, the main analyses focused on comparisons between infants for whom DHM was initiated within 24 h and those for whom DHM was initiated at 25–48 h after birth. Infants in the NRNJ cohort were used as the reference group in these analyses. Each model included the prespecified covariates described above.

In the sensitivity analyses, additional models were constructed, encompassing SGA, mode of delivery, antenatal steroid exposure, and treatment for patent ductus arteriosus (all treated as binary variables), to assess the robustness of the primary findings.

Weakly informative priors were specified as Normal (0, 2) for regression coefficients and Student-*t* (3, 0, 10) for the intercept. Markov Chain Monte Carlo sampling with the No-U-Turn Sampler, as implemented in the *brms* package in R, was used for estimating the models.

Four chains of 10,000 iterations each were run, with the first 3000 iterations used as the warm-up. Convergence was confirmed by inspection of trace plots and Gelman–Rubin statistics ($\hat{R}$ < 1.01) for all parameters. Posterior odds ratios (ORs) with 95% credible intervals (CrIs) were reported. Moreover, to quantify the probability of a beneficial effect, the posterior probability that the OR was <1 [*p* (OR < 1)] was calculated.

For the secondary outcome of age at achievement of full enteral feeding, Bayesian quantile regression (τ = 0.5) was applied for estimating the median difference across the three groups. This approach was selected because the distribution of days to full enteral feeding was right-skewed with several extreme values. Posterior median differences and their 95% CrIs were reported, alongside the posterior probability that the median difference was <0 [*p* (diff < 0)], indicating the probability that DHM initiation was associated with earlier full enteral feeding achievement.

Besides the main analysis conducted on infants with <1500 g birth weights, a prespecified subgroup analysis was performed on infants with <1000 g birth weights to investigate whether the associations between DHM initiation timing and outcomes were consistent among extremely-low-birth-weight infants.

To overcome potential temporal confounding, an additional sensitivity analysis was performed, restricting the DHM cohort to infants born in 2022 and comparing them with the NRNJ 2022 cohort. Analyses for NEC were not performed, owing to the small number of NEC events in this restricted cohort; however, Bayesian models were applied for in-hospital mortality and time to full enteral feeding.

All analyses were performed using R (version 4.1.2; R Foundation for Statistical Computing, Vienna, Austria) and the brms package.

## 3. Results

### Study Population

**Figure 1:**
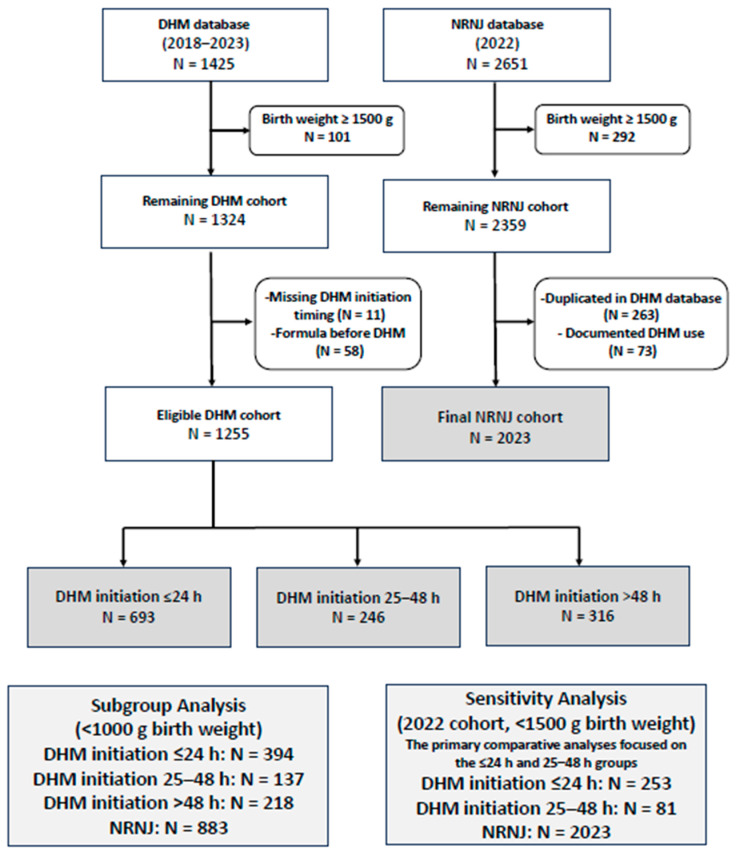
Flow diagram of the study cohort selection. Infants in the DHM database were categorized into three groups based on DHM initiation timing (≤24, 25–48, and >48 h after birth). Main analyses focus on early initiation (≤48 h), whereas infants with later initiation (>48 h) are included in descriptive analyses. NRNJ, Neonatal Research Network of Japan; DHM, donor human milk.

Overall, 1425 infants were identified in the DHM database between 2018 and 2023, and 2651 infants were registered in the NRNJ registry in 2022. After excluding infants with ≥1500 g birth weights, those with missing data on DHM initiation timing, and those who received formula before DHM, 1255 infants (across 51 participating NICUs) remained eligible for inclusion in the DHM cohort. In the NRNJ registry, after excluding infants with ≥1500 g birth weights, duplicated registrations in the DHM database, and documented DHM use, 2023 infants (across 90 participating NICUs) were included in the final comparison cohort (Figure 1).

The baseline characteristics of infants with <1500 g birth weights are summarized in Table 1.

Sex distribution was generally comparable across the groups. In contrast, gestational age, birth weight, and several clinical characteristics differed between the DHM groups and the NRNJ cohort. Baseline characteristics were generally comparable between the ≤24 h and 25–48 h groups, although differences remained in the proportions of SGA infants, antenatal steroid exposure, and treatment for patent ductus arteriosus. All groups demonstrated low NEC incidence. Among infants with <1500 g birth weights, NEC developed in 1.31% (9/686) and 1.83% (29/1583) in the ≤24 h DHM and NRNJ groups, respectively. Among infants with <1000 g birth weights, NEC developed in 2.31% and 3.67% in the ≤24 h DHM group and NRNJ cohort, respectively.

Both DHM groups demonstrated lower mortality rates. Among infants with <1500 g birth weights, mortality rates were 4.53% (31/685) and 6.23% (126/2023) in the ≤24 h DHM and NRNJ groups, respectively. Among infants with <1000 g birth weights, mortality rates were 7.24% and 11.10% in the ≤24 h DHM and NRNJ groups, respectively.

Infants receiving DHM demonstrated a tendency toward earlier full enteral feeding achievement. This trend was clearer among infants with <1000 g birth weights, for whom the median time was 11 days in both DHM groups compared with 13 (interquartile range, 9–17) days in the NRNJ cohort. Outcome data with missing values were excluded for each outcome. Therefore, the denominators for NEC, mortality, and time to full enteral feeding differed slightly across outcomes and represent only infants with available data.

Crude outcomes across all groups, including infants for whom DHM was initiated after 48 h, are presented in Appendix A Table A1.

The adjusted Bayesian estimates for NEC, mortality, and time to full enteral feeding are summarized in Table 2 (infants with <1500 g birth weights) and Table 3 (infants with <1000 g birth weights). Overall, both DHM groups exhibited lower posterior odds of mortality, a suggestive reduction in NEC—particularly when DHM was initiated at ≤24 h—and earlier full enteral feeding achievement than the NRNJ cohort. These findings were largely consistent in sensitivity analyses using fully adjusted models (Appendix A Table A2 and Table A3) and in the 2022-restricted cohort analysis for mortality and time to full enteral feeding (Appendix A Table A4). Crude outcomes for the 2022 cohort are summarized in Appendix A Table A5.

## 4. Discussion

Using a large multicenter dataset in Japan, we investigated the association between DHM initiation timing and neonatal outcomes in VLBW infants. Although the comparison cohort lacked detailed information on early nutritional exposure, earlier DHM initiation was associated with lower in-hospital mortality and earlier achievement of full enteral feeding without evidence of increased NEC risk. Therefore, the observed associations should be interpreted cautiously, as they may reflect broader differences in nutritional practices rather than the effect of DHM initiation timing alone. However, the effects of DHM initiation timing could not be clearly distinguished from broader early enteral feeding practices, availability of the mother’s own milk, illness severity, clinical condition, institutional protocols, or differences in NICU care quality. In addition, several clinically important variables, including respiratory support, infection status, antibiotic exposure, and institutional feeding protocols, were not consistently available across the registries, limiting further adjustment for potential confounding factors.

In-hospital mortality exhibited a more consistent reduction than NEC, with comparable trends observed in both <1500 g and <1000 g infants. The Bayesian analysis demonstrated a high posterior probability that DHM initiation was associated with reduced mortality. By contrast, although the early DHM group demonstrated lower NEC incidence, the overall event rate was low, and credible intervals were wide, indicating that this finding should be interpreted as suggestive rather than definitive. Nevertheless, this observation is broadly consistent with previous evidence suggesting that early human milk–based feeding is not associated with an increased risk of NEC in preterm infants.

Additional analyses supported the robustness of the primary findings. Sensitivity analyses, additionally adjusting for perinatal and clinical factors, including SGA, mode of delivery, antenatal steroid exposure, and treatment for patent ductus arteriosus, revealed largely unchanged direction and magnitude of associations. Moreover, analyses restricted to the 2022 cohort demonstrated consistent results in terms of direction for mortality and time to full enteral feeding, despite the limited number of NEC events. Furthermore, crude outcome comparisons exhibited comparable trends, supporting the overall consistency of the findings across different analytical approaches.

Our findings should be interpreted in the context of previous literature on early enteral feeding. Randomized trials and systematic reviews, frequently defining early feeding as initiation within 24–72 h after birth, have generally indicated that early feeding is associated with reduced mortality and sepsis and shorter parenteral nutrition duration, while inconsistently demonstrating significant reductions in NEC incidence. Notably, these studies have collectively supported that early enteral feeding does not elevate the risk of NEC or mortality [3,4,5,13,17,18]. Our findings align with this body of evidence and further extend it by specifically focusing on DHM initiation timing.

This study represents one of the first large multicenter analyses investigating the association between early DHM initiation timing and neonatal outcomes using registry-based timing data and a Bayesian analytical framework. By categorizing DHM exposure into ≤24, 25–48, and >48 h, with the NRNJ cohort as the reference group, we evaluated associations across clinically relevant DHM initiation timing categories. Additionally, using Bayesian modeling facilitated the estimation of posterior probabilities for rare but clinically significant outcomes, including mortality and time to full enteral feeding, particularly in ELBW infants, a population in which such quantitative estimates have been limited.

In the subgroup of infants with <1000 g birth weights, our findings suggest a high likelihood that early DHM initiation is associated with lower mortality and earlier achievement of full enteral feeding. This finding offers ELBW-specific quantitative evidence complementing those of previous studies, which have mainly demonstrated reductions in sepsis but have inconsistently exhibited changes in NEC. These results support the safety of early human milk–based feeding even in this highest-risk population; however, residual confounding cannot be excluded.

Regarding enteral feeding outcomes, infants in the DHM group achieved full enteral feeding earlier than those in the NRNJ cohort. Although the absolute difference in time to full feeding was modest, even a 1–2-day reduction may be clinically meaningful, as it could reduce the duration of parenteral nutrition and associated risks of infection. These findings align with those of previous studies demonstrating that earlier enteral feeding facilitates feeding tolerance and reduces prolonged parenteral nutrition-related complications.

Several biological mechanisms may explain these findings. Human milk contains a wide range of bioactive components, including human milk oligosaccharides, growth factors, immunoglobulins, and anti-inflammatory cytokines, which promote intestinal maturation, enhance mucosal barrier function, and modulate immune responses [19,20,21]. Early exposure to these components during the critical postnatal period may facilitate intestinal adaptation and reduce inflammatory injury, thereby lowering susceptibility to NEC. Furthermore, earlier establishment of enteral feeding reduces reliance on parenteral nutrition, which is associated with an increased risk of infection and metabolic complications [4,14,22,23]. However, the mechanisms proposed in the present study remain speculative given the lack of detailed information regarding DHM composition, pasteurization effects, feeding volume, and the proportion of the mother’s own milk.

In Japan, clinical practices may vary across institutions, as no standardized national guidelines exist regarding enteral feeding initiation timing or DHM use. Considering that human milk bank systems were only introduced relatively recently, in 2017, such variability may persist. In this context, our findings offer crucial real-world evidence on DHM initiation timing and its potential influence on neonatal outcomes.

This study had several limitations. First, comprehensive information on early nutritional practices was not available in the NRNJ cohort. Infants in this group potentially received heterogeneous nutritional exposures, including their mother’s own milk, formula, or delayed enteral feeding. Therefore, the observed associations may reflect differences in nutritional exposure type and timing rather than the effect of DHM alone. Second, unmeasured institutional differences between centers using DHM and those that did not could not be fully accounted for, potentially impacting the observed associations. Third, temporal differences between datasets may introduce temporal confounding, as the DHM registry covered 2018–2023, whereas the comparison cohort was derived from 2022 data only; however, sensitivity analyses restricted to the 2022 cohort revealed largely consistent findings. Fourth, missing outcome data, particularly for NEC occurrence and timing to full enteral feeding in the NRNJ registry, may have influenced the results. Fifth, infants for whom DHM was initiated after 48 h were analyzed as a clinically distinct group, potentially introducing selection bias; the absence of comprehensive data on pre-DHM feeding practices limited the interpretability of outcomes in this group. Finally, as this was an observational study, residual confounding and confounding by indication in feeding decisions could not be excluded.

## 5. Conclusions

Early DHM initiation may be associated with lower mortality and earlier achievement of full enteral feeding in VLBW infants, without clear evidence of an elevated NEC risk. However, because detailed feeding information was unavailable in the comparison cohort, these findings should be interpreted cautiously and may reflect broader differences in early nutritional practices and institutional feeding strategies rather than the effect of DHM initiation timing alone. Further prospective studies are warranted.

## Figures and Tables

**Table 1:** Baseline characteristics of VLBW (<1500 g) infants in the DHM and NRNJ groups.

	DHM (≤24 h) N = 693	DHM (25–48 h) N = 246	DHM (>48 h) N = 316	NRNJ N = 2023	SMD vs. NRNJ (≤24 h/25–48 h/>48 h)	SMD (≤24 h vs. 25–48 h)
Female, *n* (%)	324 (50.7)	123 (50.0)	162 (51.3)	993 (49.1)	0.05/0.02/0.04	0.07
Birth weight, g, median (IQR)	943 (708–1215)	964 (707–1210)	868 (656–1095)	1070 (780–1310)	0.26/0.25/0.50	0.01
Gestational age, days, median (IQR)	197 (180–212)	198 (182–216)	192 (178–205)	204 (187–219)	0.28/0.16/0.48	0.12
Apgar score at 1 min, median (IQR)	4 (2–6)	5 (3–6)	4 (2–6)	5 (3–7)	0.30/0.16/0.33	0.14
Apgar score at 5 min, median (IQR)	7 (6–8)	7 (6–8)	7 (6–8)	8 (6–9)	0.12/0.07/0.19	0.05
Small for gestational age, *n* (%)	268 (61.3)	119 (51.6)	130 (58.2)	909 (55.0)	0.20/0.06/0.13	0.20
Cesarean section, *n* (%)	494 (85.3)	159 (85.5)	243 (90.0)	1434 (84.8)	0.02/0.02/0.15	<0.01
Antenatal steroid exposure, *n* (%)	475 (68.5)	120 (48.8)	182 (57.6)	1181 (70.8)	0.05/0.47/0.28	0.41
Treatment for patent ductus arteriosus, *n* (%)	190 (27.4)	48 (19.5)	94 (29.7)	581 (36.7)	0.20/0.36/0.14	0.18

Data are presented as medians (IQRs) or numbers (percentages), as appropriate. A small-for-gestational-age infant is defined as having a birth weight below the 10th percentile for gestational age and sex. SMDs are calculated to quantify group differences between the DHM and NRNJ groups; values < 0.10 are considered negligible. Abbreviations: VLBW, very low birth weight; DHM, donor human milk; NRNJ, Neonatal Research Network of Japan; IQRs, interquartile ranges; SMDs, standardized mean differences.

**Table 2:** Bayesian estimates for NEC, mortality, and time to full enteral feeding according to DHM initiation timing in infants with <1500 g birth weights.

Outcome	Comparison	Median OR/Diff	95% CrI	Probability
NEC	≤24 h vs. NRNJ	0.54	0.24–1.10	*p* (OR < 1) = 0.96
	25–48 h vs. NRNJ	1.02	0.35–2.53	0.48
	≤24 h vs. 25–48 h	0.53	0.18–1.72	0.87
Mortality	≤24 h vs. NRNJ	0.50	0.32–0.76	*p* (OR < 1) = 0.99
	25–48 h vs. NRNJ	0.55	0.26–1.02	0.97
	≤24 h vs. 25–48 h	0.93	0.45–2.04	0.57
Time to full enteral feeding	≤24 h vs. NRNJ	−1.46 days	−1.87 to −1.05	*p* (diff < 0) = 0.99
	25–48 h vs. NRNJ	−0.72 days	−1.34 to −0.04	0.98
	≤24 h vs. 25–48 h	−0.73 days	−1.45 to −0.07	0.99

Posterior ORs (for NEC and mortality) and median differences in days (for full enteral feeding), with 95% CrIs, are estimated from Bayesian logistic and quantile regression models adjusted for sex, gestational age, birth weight, and 1 min Apgar score. *p* (OR < 1) and *p* (diff < 0) represent posterior probabilities of beneficial associations and do not correspond to frequentist *p*-values. Abbreviations: NEC, necrotizing enterocolitis; DHM, donor human milk; NRNJ, Neonatal Research Network of Japan; OR, odds ratio; CrIs, credible intervals.

**Table 3:** Bayesian estimates for NEC, mortality, and time to full enteral feeding according to DHM initiation timing in infants with <1000 g birth weights.

Outcome	Comparison	Median OR/Diff	95% CrI	Probability
NEC	≤24 h vs. NRNJ	0.59	0.26–1.23	*p* (OR < 1) = 0.92
	25–48 h vs. NRNJ	1.14	0.38–2.89	0.40
	≤24 h vs. 25–48 h	0.52	0.17–1.74	0.87
Mortality	≤24 h vs. NRNJ	0.54	0.34–0.86	*p* (OR < 1) = 0.99
	25–48 h vs. NRNJ	0.58	0.27–1.16	0.94
	≤24 h vs. 25–48 h	0.93	0.43–2.19	0.56
Time to full enteral feeding	≤24 h vs. NRNJ	−2.22 days	−2.99 to −1.44	*p* (diff < 0) = 0.99
	25–48 h vs. NRNJ	−1.30 days	−2.54 to −0.06	0.98
	≤24 h vs. 25–48 h	−0.93 days	−2.19 to 0.37	0.92

Posterior ORs (for NEC and mortality) and median differences in days (for full enteral feeding), with 95% CrIs, are estimated from Bayesian logistic and quantile regression models adjusted for sex, gestational age, birth weight, and 1 min Apgar score. *p* (OR < 1) and *p* (diff < 0) represent posterior probabilities of beneficial associations and do not correspond to frequentist *p*-values. Abbreviations: NEC, necrotizing enterocolitis; NRNJ, Neonatal Research Network of Japan; OR, odds ratio; CrIs, credible intervals.

## Data Availability

The data presented in this study are available on request from the corresponding author due to privacy and ethical restrictions related to the use of registry data.

## Abbreviations

The following abbreviations are used in this manuscript:

DHM	Donor human milk
NRNJ	Neonatal Research Network of Japan
OR	Odds ratio
SMD	Standardized mean difference
VLBW	Very low birth weight

## Appendix A

**Table A1:** Crude outcomes according to DHM initiation timing and NRNJ cohort.

<1500 g	DHM (≤24 h) N = 693	DHM (25–48 h) N = 246	DHM (>48 h) N = 316	NRNJ N = 2023
NEC, *n* (%)	9 (1.31)	5 (2.06)	3 (0.96)	29 (1.83)
Missing, *n*	7	3	3	440
Mortality, *n* (%)	31 (4.53)	11 (4.53)	8 (2.56)	126 (6.23)
Missing, *n*	8	3	4	0
Time to full enteral feeding, days (IQR)	9 (7–12)	9 (7–12)	13 (10–19)	9 (7–14)
Missing, *n*	20	9	6	483
**<1000 g**	**DHM (≤24 h)** **N = 394**	**DHM (25–48 h)** **N = 137**	**DHM (>48 h)** **N = 218**	**NRNJ** **N = 883**
NEC, *n* (%)	9 (2.31)	5 (3.68)	2 (0.93)	25 (3.67)
Missing, *n*	5	1	2	202
Mortality, *n* (%)	28 (7.24)	10 (7.41)	6 (2.76)	98 (11.10)
Missing, *n*	7	2	1	0
Time to full enteral feeding, days (IQR)	11 (8–14)	11 (9–15)	14 (11–19)	13 (9–17)
Missing, *n*	16	6	6	240

**Table A2:** Bayesian estimates for NEC, mortality, and time to full enteral feeding according to DHM initiation timing in infants with <1500 g birth weights, with additional adjustment for perinatal and clinical covariates.

Outcome	Comparison	Median OR/Diff	95% CrI	Probability
NEC	≤24 h vs. NRNJ	0.52	0.22–1.14	*p* (OR < 1) = 0.95
	25–48 h vs. NRNJ	1.36	0.44–3.48	0.28
	≤24 h vs. 25–48 h	0.38	0.12–1.36	0.94
Mortality	≤24 h vs. NRNJ	0.55	0.33–0.88	*p* (OR < 1) = 0.99
	25–48 h vs. NRNJ	0.49	0.21–1.02	0.97
	≤24 h vs. 25–48 h	1.13	0.50–2.79	0.39
Time to full enteral feeding	≤24 h vs. NRNJ	−1.25 days	−1.66 to −0.85	*p* (diff < 0) = 0.99
	25–48 h vs. NRNJ	−0.51 days	−1.12 to 0.17	0.94
	≤24 h vs. 25–48 h	−0.73 days	−1.45 to −0.11	0.99

Posterior ORs (for NEC and mortality) and median differences in days (for full enteral feeding) with 95% CrIs are estimated using Bayesian logistic and quantile regression models. Models are adjusted for sex, gestational age, birth weight, and 1 min Apgar score, and additionally for small-for-gestational-age status, mode of delivery (cesarean section), antenatal steroid exposure, and treatment for patent ductus arteriosus. *p* (OR < 1) and *p* (diff < 0) represent posterior probabilities of beneficial associations and do not correspond to frequentist *p*-values. Abbreviations: NEC, necrotizing enterocolitis; DHM, donor human milk; NRNJ, Neonatal Research Network of Japan; OR, odds ratio; CrIs, credible intervals.

**Table A3:** Bayesian estimates for NEC, mortality, and time to full enteral feeding based on DHM initiation timing in infants with <1000 g birth weights, with additional adjustment for perinatal and clinical covariates.

Outcome	Comparison	Median OR/Diff	95% CrI	Probability
NEC	≤24 h vs. NRNJ	0.57	0.24–1.27	*p* (OR < 1) = 0.91
	25–48 h vs. NRNJ	1.48	0.47–3.89	0.24
	≤24 h vs. 25–48 h	0.38	0.12–1.36	0.93
Mortality	≤24 h vs. NRNJ	0.57	0.33–0.93	*p* (OR < 1) = 0.99
	25–48 h vs. NRNJ	0.55	0.24–1.19	0.94
	≤24 h vs. 25–48 h	1.02	0.44–2.51	0.48
Time to full enteral feeding	≤24 h vs. NRNJ	−1.82 days	−2.61 to −1.04	*p* (diff < 0) = 0.99
	25–48 h vs. NRNJ	−0.98 days	−2.14 to 0.30	0.94
	≤24 h vs. 25–48 h	−0.81 days	−2.16 to 0.35	0.91

Posterior ORs (for NEC and mortality) and median differences in days (for full enteral feeding) with 95% CrIs are estimated using Bayesian logistic and quantile regression models. Models are adjusted for sex, gestational age, birth weight, and 1 min Apgar score, and additionally for small-for-gestational-age status, mode of delivery (cesarean section), antenatal steroid exposure, and treatment for patent ductus arteriosus. *p* (OR < 1) and *p* (diff < 0) represent posterior probabilities of beneficial associations and do not correspond to frequentist *p*-values. Abbreviations: NEC, necrotizing enterocolitis; DHM, donor human milk; NRNJ, Neonatal Research Network of Japan; OR, odds ratio; CrIs, credible intervals.

**Table A4:** Bayesian estimates for mortality and time to full enteral feeding based on DHM initiation timing in infants with <1500 g birth weights in the 2022 cohort.

Outcome	Comparison	Median OR/Diff	95% CrI	Probability
Mortality	≤24 h vs. NRNJ	0.29	0.12–0.61	*p* (OR < 1) = 0.99
	25–48 h vs. NRNJ	0.47	0.12–1.32	0.92
	≤24 h vs. 25–48 h	0.62	0.16–2.84	0.74
Time to full enteral feeding	≤24 h vs. NRNJ	−1.78 days	−2.40 to −1.15	*p* (diff < 0) = 0.99
	25–48 h vs. NRNJ	−0.89 days	−1.93 to 0.16	0.95
	≤24 h vs. 25–48 h	−0.89 days	−2.04 to 0.26	0.94

Posterior ORs for mortality and median differences in days for full enteral feeding with 95% CrIs are estimated using Bayesian logistic and quantile regression models. Models are adjusted for sex, gestational age, birth weight, and 1 min Apgar score. Analyses for NEC are not performed owing to the small number of NEC events in the 2022 cohort. *p* (OR < 1) and *p* (diff < 0) indicate the posterior probability of a beneficial association. Abbreviations: DHM, donor human milk; NRNJ, Neonatal Research Network of Japan; OR, odds ratio; CrI, credible intervals.

**Table A5:** Crude outcomes based on DHM initiation timing in infants with <1500 g birth weights in the 2022 cohort.

Outcomes	DHM (≤24 h) N = 253	DHM (25–48 h) N = 81	NRNJ N = 2023
NEC, *n* (%)	2 (0.79)	0 (0)	29 (1.83)
Missing, *n*	0	1	440
Mortality, *n* (%)	7 (2.81)	3 (3.75)	126 (6.23)
Missing, *n*	4	1	0
Time to full enteral feeding, days (IQR)	9 (7–12)	9 (7–13)	9 (7–14)
Missing, *n*	3	1	483

Data are presented as *n* (%) or medians (interquartile ranges). Percentages are calculated on the basis of available data for each outcome.
